# Supplementary material for: Cost-Effective Synthesis of Efficient CoWO4/Ni Nanocomposite Electrode Material for Supercapacitor Applications
Source: Nanomaterials (Basel). 2020 Nov 4;10(11):2195. doi: 10.3390/nano10112195 (PMC7692640; doi:10.3390/nano10112195)
Supplement: Supplementary file 1 [file nanomaterials-10-02195-s001.pdf]

Supporting Information

Cost-Effective Synthesis of Efficient $CoWO_4$/Ni Nanocomposite Electrode Material for Supercapacitor Applications

Kannadasan Thiagarajan [1], Dhandapani Balaji [1], Jagannathan Madhavan [1,*], Jayaraman Theerthagiri [2], Seung Jun Lee [2], Ki-Young Kwon [2,*] and Myong Yong Choi [2,*]

1 Solar Energy Lab, Department of Chemistry, Thiruvalluvar University, Vellore 632 115, India; kthiyagarajanmphil6@gmail.com (K.T.); baladgp@gmail.com (D.B.)

2 Department of Chemistry and Research Institute of Natural Sciences, Gyeongsang National University, Jinju 52828, Korea; j.theerthagiri@gmail.com (J.T.); venus272@gnu.ac.kr (S.J.L.)

* Correspondence: jagan.madhavan@gmail.com (J.M.); kykwon@gnu.ac.kr (K.-Y.K.); mychoi@gnu.ac.kr (M.Y.C.)

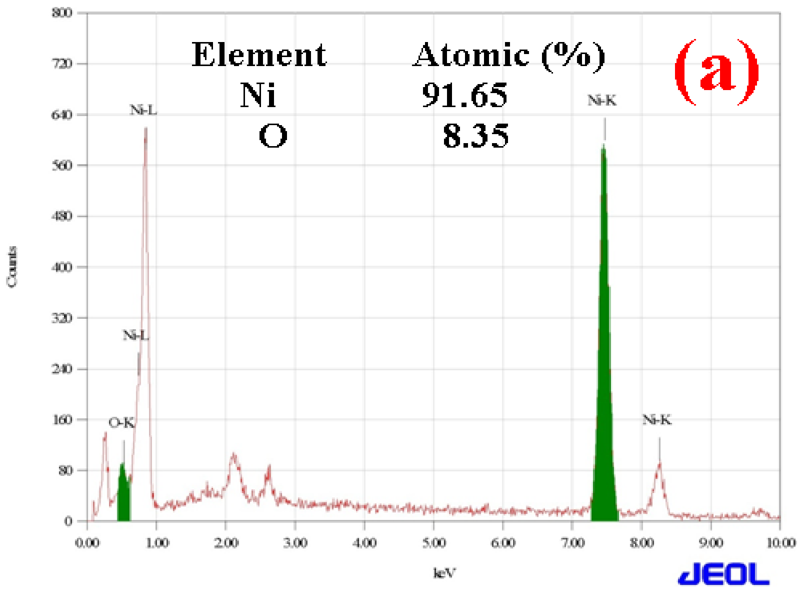

(a) Ni nanoparticles

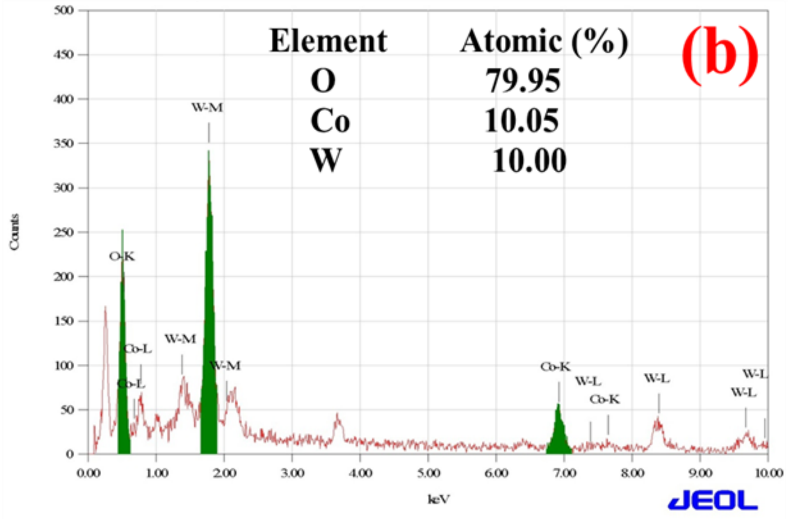

(b) Bare CWO

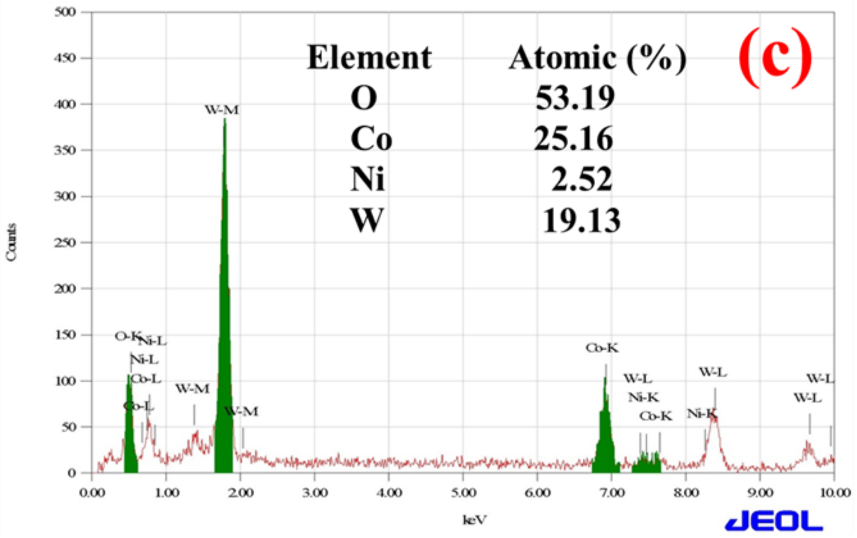

(c) CWO-Ni3 composite

Figure S1: EDAX spectrum of (**a**) Ni nanoparticles, (**b**) bare CWO, and (**c**) CWO-Ni3 composite.
